# Toward a biopsy-free approach: cardiac magnetic resonance T1 mapping for detecting heart transplant rejection

**DOI:** 10.1093/ehjci/jeag080

**Published:** 2026-03-26

**Authors:** Marko Taipale, Markku Pentikäinen, Laura Martelius, Aino Mutka, Soili Kytölä, Matti Kankainen, Juha I Peltonen, Simo Syrjälä, Arttu Lahtiharju, Jyri Lommi, Timo Jahnukainen, Karl Lemström, Tiina Ojala

**Affiliations:** Department of Pediatric Cardiology, Pediatric Research Center, University of Helsinki and Helsinki University Hospital, PO Box 281, Stenbäckinkatu 11, Helsinki FI-00029 HUS, Finland; Department of Cardiology, Heart and Lung Center, Helsinki University Hospital and University of Helsinki, Helsinki, Finland; Department of Radiology, Helsinki University Hospital and University of Helsinki, Helsinki, Finland; Department of Pathology, Helsinki University Hospital and University of Helsinki, Helsinki, Finland; Laboratory of Genetics, HUS Diagnostic Center, Helsinki University Hospital and University of Helsinki, Helsinki, Finland; Laboratory of Genetics, HUS Diagnostic Center, Helsinki and Uusimaa Hospital District, and Hematology Research Unit Helsinki, Translational Immunology Research Program, University of Helsinki, Helsinki, Finland; Department of Radiology, Helsinki University Hospital and University of Helsinki, Helsinki, Finland; Department of Cardiothoracic Surgery, Heart and Lung Center, Helsinki University Hospital and University of Helsinki, Helsinki, Finland; Department of Cardiothoracic Surgery, Heart and Lung Center, Helsinki University Hospital and University of Helsinki, Helsinki, Finland; Department of Cardiology, Heart and Lung Center, Helsinki University Hospital and University of Helsinki, Helsinki, Finland; Department of Pediatric Nephrology and Transplantation, Helsinki University Hospital and University of Helsinki, Helsinki, Finland; Department of Cardiothoracic Surgery, Heart and Lung Center, Helsinki University Hospital and University of Helsinki, Helsinki, Finland; Department of Pediatric Cardiology, Pediatric Research Center, University of Helsinki and Helsinki University Hospital, PO Box 281, Stenbäckinkatu 11, Helsinki FI-00029 HUS, Finland

**Keywords:** CMR, T1 mapping, T2 mapping, heart transplantation, EMB, dd-cfDNA

Clinical Trial Registration

ClinicalTrials.gov Identifier: NCT04311346.

Cardiac magnetic resonance (CMR) T1 and T2 mapping, indicators of myocardial inflammation and oedema, show promise as a non-invasive approach to assess acute rejection in heart transplants.^1–3^ This study evaluated the diagnostic accuracy of CMR mapping models in detecting acute rejection, and compared their performance with endomyocardial biopsy (EMB), clinical data, and donor-derived cell-free DNA (dd-cfDNA).

This blinded prospective study (Trial NCT04311346) was conducted at the paediatric and adult centers of Helsinki University Hospital. The research protocol was approved by the local ethics committee (HUS; HUS/3341/2019). Acute rejection at each time point was defined by the presence of at least two of the following three criteria: (1) EMB showing grade ≥2R acute cellular rejection or grade ≥1 antibody-mediated rejection; (2) clinical signs or echocardiographic evidence of rejection; and (3) dd-cfDNA >0.20%. If one criterion was unavailable, rejection was diagnosed when one of the remaining two criteria was met. EMBs were evaluated using ISHLT criteria, and dd-cfDNA was measured by droplet digital PCR.

We blindly analyzed CMR studies from paediatric and adult heart transplant patients 1–24 months post-transplant, including five additional paediatric acute rejection episodes occurring 3–14 years after transplantation.

All CMR scans were conducted on 1.5T MRI systems. T1 and T2 mapping were performed in three short-axis planes using the MOLLI sequence for T1, GraSE for paediatric T2, and T2-prepared bSSFP for adult T2 mapping. T1 and T2 relaxation times were calculated across all 16 myocardial segments. Vendor-specific T1 and T2 mapping reference values were established for paediatric and adult patients due to baseline differences observed in phantom and patient studies.^4^ The detailed methods are described in our previous study.^4^

To assess myocardial involvement during rejection, we applied three CMR T1 and T2 mapping models (*Figure 1*), including two global models (models 1 and 2) and one conventional mid-septal model (model 3). Model 1, the global segment-count model, classified each myocardial segment as abnormal if its T1 or T2 value exceeded a statistically defined cutoff and recorded the total number of abnormal segments. Model 2, the global mean model, calculated the average T1 or T2 relaxation time across all 16 myocardial segments. Model 3, the conventional mid-septal model, calculated the mean T1 or T2 value from the two mid-septal segments (segments 8 and 9). Cutoff values for all three models were determined by ROC analysis using the Youden index.

**Figure 1:**
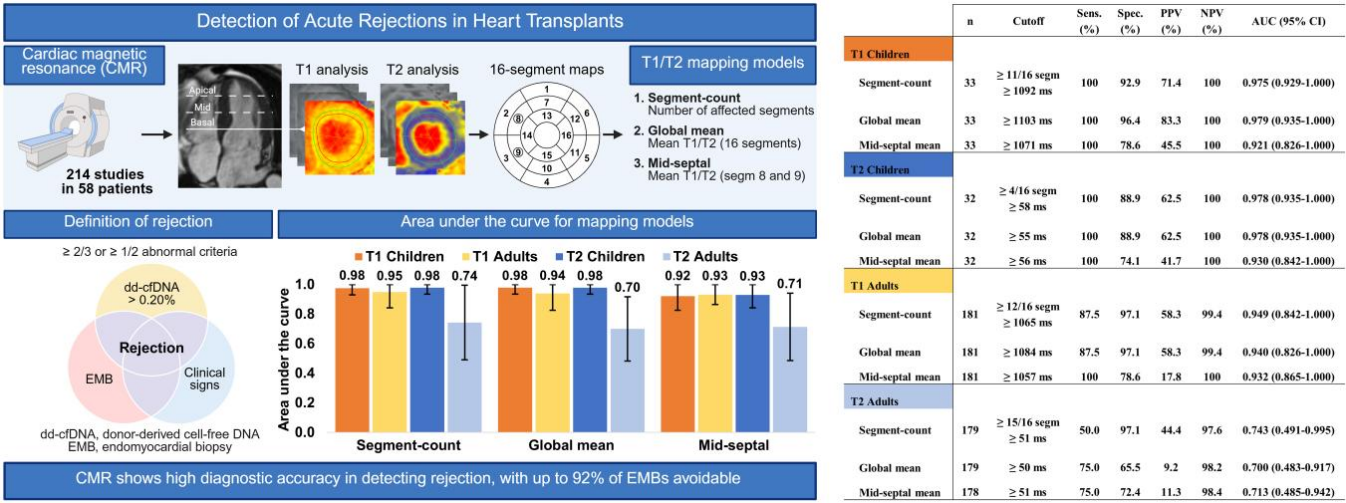
CMR T1 and T2 mapping methodology and diagnostic performance for detecting acute heart transplant rejection. Optimal cutoff values for each mapping model were determined by receiver operating characteristic (ROC) analysis using the Youden index. Segment-count indicates the number of myocardial segments exceeding the cutoff value; global mean, the mean T1/T2 value across all 16 segments; and mid-septal mean, the mean T1/T2 value of the two mid-septal segments (segments 8 and 9). AUC, area under the curve; CI, confidence interval; NPV, negative predictive value; PPV, positive predictive value.

Among 214 CMR studies conducted in 58 patients, 13 cases of acute rejection were identified, including five in four paediatric patients and eight in five adults (see Supplementary data online, *Figure S1* and *Table S1*).

All three T1 mapping models—segment-count, global mean, and mid-septal—demonstrated excellent diagnostic performance in detecting acute rejection; however, the mid-septal model showed lower specificity (*Figures 1* and *S2*). In children, the global models achieved 100% sensitivity and 93–96% specificity; the mid-septal model, 100% and 79%. In adults, the global models showed 88% sensitivity and 97% specificity; the mid-septal model, 100% and 79%. T2 values showed high diagnostic accuracy only in paediatric patients.

If used as a first-line screening tool, CMR-based surveillance could have avoided 195 of 214 EMBs (91%) with the segment-count model, 196 (92%) with the global mean model, and 158 (74%) with the mid-septal model, assuming that all CMR-positive cases were confirmed by EMB.

Importantly, models based on isolated rejection criteria (EMB, clinical signs, or dd-cfDNA alone) were less accurate than models using the full three-parameter composite definition, particularly in adults (see Supplementary data online, *Table S2*).

In this prospective study involving both paediatric and adult heart transplant recipients, we demonstrate that CMR provides excellent diagnostic accuracy for detecting acute transplant rejection. Although previous studies have also highlighted the diagnostic potential of CMR for transplant rejection,^1–3^ their findings have been limited by a focus on the mid-septal myocardium or by the absence of a comprehensive reference standard. Our results show that T1-based global models—the segment-count and global mean approaches, covering around 40% of the myocardial mass—can reliably identify rejection with high sensitivity and specificity across age groups. These findings support CMR as a promising non-invasive screening tool with the potential to serve as a virtual biopsy.

A key clinical implication of our findings is the potential to dramatically reduce the need for routine EMBs. Given the low incidence of rejection, the diagnostic limitations^5^ and procedural risks associated with EMB, and the high number needed to test with routine EMB, accurate non-invasive tools such as CMR are needed. Avoiding routine EMB would therefore represent a significant advancement in transplant care. In the future, patients with positive CMR findings could be further evaluated using a combination of dd-cfDNA analysis and clinical data, potentially limiting the need for EMB to cases where these non-invasive methods raise concern or the clinical picture remains inconclusive.

Notably, while T1 mapping consistently demonstrated high diagnostic accuracy, T2 mapping was effective in paediatric patients but underperformed in adults. This may reflect the higher rate of more severe rejection episodes among affected children, possibly related to non-adherence to immunosuppressive therapy.

Our study has several limitations. It was conducted at a single centre, dd-cfDNA samples were unavailable at the 24-month post-transplant time point, different imaging vendors were used for paediatric and adult patients, and acute rejection was present in only 6% (13/214) of CMR studies.

In conclusion, across both global 16-segment models, CMR T1 mapping demonstrated excellent diagnostic accuracy for detecting acute rejection in paediatric and adult heart transplant recipients. Combined with clinical data and dd-cfDNA, CMR T1 mapping may provide a robust alternative to biopsy and reduce reliance on routine biopsy in post-transplant surveillance.

## Supplementary Material

jeag080_Supplementary_Data

## Data Availability

The data underlying this article cannot be shared publicly due to ethical reasons. The data is available for review by onsite visitors.
