# Supplementary material for: Toward a biopsy-free approach: cardiac magnetic resonance T1 mapping for detecting heart transplant rejection
Source: Eur Heart J Cardiovasc Imaging. 2026 Mar 26;27(6):1284–6. doi: 10.1093/ehjci/jeag080 (PMC13222716; doi:10.1093/ehjci/jeag080)
Supplement: jeag080_Supplementary_Data [file jeag080_supplementary_data.docx]

**Supplementary data**

|  | **Title** | **Page** |
| --- | --- | --- |
| **Figure S1** | **Rejection events and isolated rejection marker findings in the study cohort.** | **2** |
| **Figure S2** | **Flow diagram of CMR studies.** | **3** |
| **Table S1** | **Baseline patient characteristics** | **4** |
| **Table S2** | **Diagnostic Accuracy of T1 and T2 to Detect Acute Rejection defined by EMB, dd-cfDNA, and clinical data** | **5** |

**FIGURE S1**

**
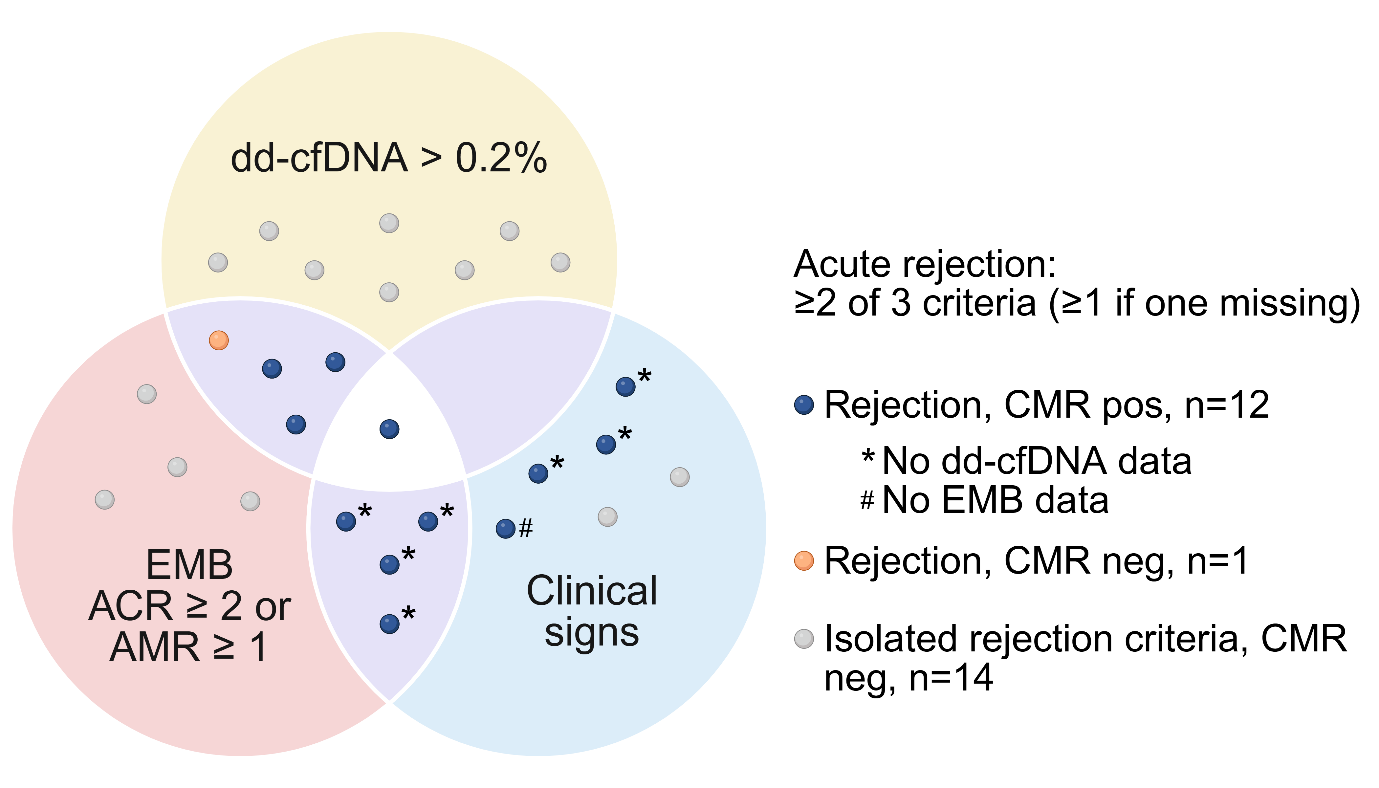
**

Rejection events and isolated rejection marker findings in the study cohort.

Acute rejection was defined as ≥2 of 3 criteria, or ≥1 when only two were available: 1) EMB showing ACR ≥ 2R or pAMR ≥ 1; 2) clinical signs or ultrasound evidence of rejection; or 3) dd-cfDNA levels > 0.20%. The diagnostic performance of all three CMR T1 mapping models was assessed against this composite reference criteria.

ACR = acute cellular rejection; AMR = antibody-mediated rejection; CMR = cardiac magnetic resonance imaging; dd-cfDNA = donor-derived cell-free DNA; EMB = endomyocardial biopsy

**FIGURE S2**

**
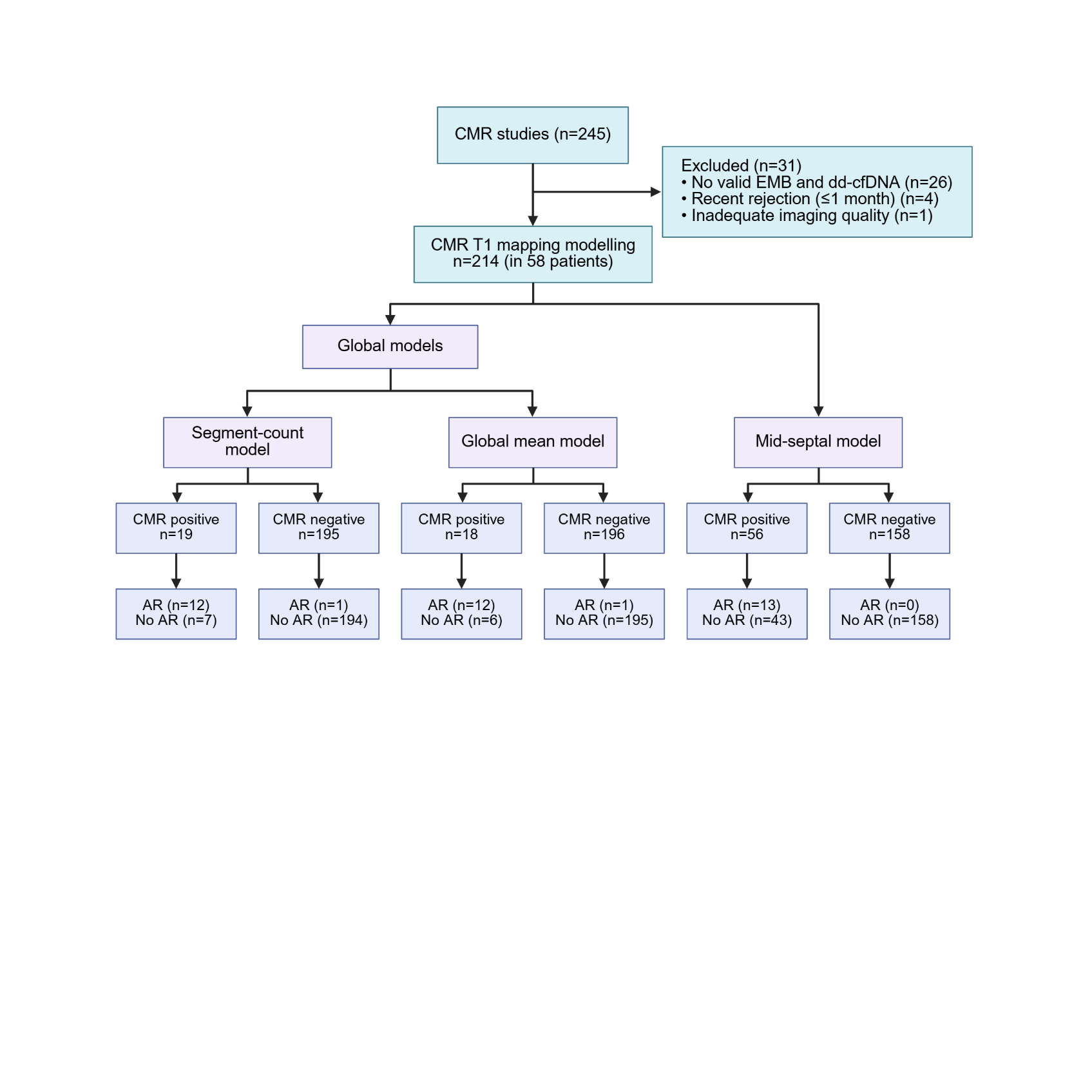
**

Flow diagram of CMR studies.

AR = acute rejection; CMR = cardiac magnetic resonance imaging; dd-cfDNA = donor-derived cell-free DNA; EMB = endomyocardial biopsy

**Table S1 Baseline patient characteristics**

|  | **Pediatric Transplants**  **n = 17** | **Adult Transplants**  **n = 41** |
| --- | --- | --- |
| Sex, male | 7 (41%) | 33 (80%) |
| Age at HTx (yrs) | 12.3 (10.3-13.3) | 54.7 (45.2-61.7) |
| BMI at HTx (kg/m2)  ISO-BMI | 19.7 (17.4-26.9) | 25.5 (22.5-27.7) |
| Donor age (yrs) | 19 (14-23) | 38 (31-46) |
| Ischemic time (min) | 205 (146-251) | 186 (117-214) |
| No. of scans per patient | 1 (1-3) | 4 (3-6) |
| CMR, mean time from HTx (mo)* | 20.2 (7.5-27.8) | - 1. (3.9-5.6) |

Values are median (interquartile range), or n (%) unless otherwise stated.

*Includes CMR studies from later time points, which correspond to rejection cases in the pediatric group.

BMI = body mass index; CMR = cardiac magnetic resonance imaging; HTx = heart transplantation; ISO-BMI = age- and sex-adjusted body mass index.

**Table S2 Diagnostic Accuracy of T1 and T2 to Detect Acute Rejection defined by EMB, dd-cfDNA, and clinical data**

|  | **n** | **Comparison** | **Sensitiv-ity (%)** | **Specific-ity (%)** | **PPV (%)** | **NPV (%)** |
| --- | --- | --- | --- | --- | --- | --- |
| **Children** |  |  |  |  |  |  |
| T1 ≥ 11/16 segm ≥ 1092 ms | 31 | EMB | 100 | 89.3 | 50.0 | 100 |
| T1 ≥ 11/16 segm ≥ 1092 ms | 33 | Clinical | 83.3 | 92.6 | 71.4 | 96.2 |
| T2 ≥ 4/16 segm ≥ 58 ms | 30 | EMB | 100 | 88.9 | 50.0 | 100 |
| T2 ≥ 4/16 segm ≥ 58 ms | 32 | Clinical | 83.3 | 88.5 | 62.5 | 95.8 |
| **Adults** |  |  |  |  |  |  |
| T1 ≥ 12/16 segm ≥ 1065 ms | 175 | EMB | 50.0 | 95.8 | 41.7 | 96.9 |
| T1 ≥ 12/16 segm ≥ 1065 ms | 157 | dd-cfDNA | 30.8 | 97.2 | 50.0 | 94.0 |
| T1 ≥ 12/16 segm ≥ 1065 ms | 181 | Clinical | 80.0 | 95.5 | 33.3 | 99.4 |
| T2 ≥ 15/16 segm ≥ 51 ms | 173 | EMB | 30.0 | 96.3 | 33.3 | 95.7 |
| T2 ≥ 15/16 segm ≥ 51 ms | 155 | dd-cfDNA | 15.4 | 97.2 | 33.3 | 92.6 |
| T2 ≥ 15/16 segm ≥ 51 ms | 179 | Clinical | 40.0 | 96.0 | 22.2 | 98.2 |

Cutoff values from segment-count model. Inadequate number of dd-cfDNA samples in children.

dd-cfDNA = donor-derived cell-free DNA; EMB = endomyocardial biopsy; NPV = negative predictive value; PPV = positive predictive value.
